# Salivary microflora and mode of delivery: a prospective case control study

**DOI:** 10.1186/s12903-015-0142-3

**Published:** 2015-12-03

**Authors:** Katarina Boustedt, Josefine Roswall, Gunnar Dahlén, Jovanna Dahlgren, Svante Twetman

**Affiliations:** Maxillofacial Unit, Halland Hospital, SE-30185 Halmstad, Sweden; Department of Pediatrics, The Sahlgrenska Academy, University of Gothenburg, Box 400, SE-40530 Gothenburg, Sweden; Department of Pediatrics, Halland Hospital, SE-30185 Halmstad, Sweden; Department of Odontology, The Sahlgrenska Academy, University of Gothenburg, Box 400, SE-40530 Gothenburg, Sweden; Department of Odontology, Faculty of Health and Medical Sciences, University of Copenhagen, Nörre Allé 20, Copenhagen N, Denmark

**Keywords:** C-section, Infants, Oral bacteria, Oral colonization, Saliva

## Abstract

**Background:**

Previous cross-sectional studies have suggested that the mode of delivery can influence the composition of oral microflora. The aim of this prospective study was to compare the salivary colonization in vaginally delivered children with children delivered by Caesarian section (C-section) during their first 6 months of life.

**Methods:**

The study group consisted of 149 consecutively enrolled infants, delivered either vaginally (*n* = 96) or by C-section (*n* = 53) that volunteered after consent of their parents. Saliva samples were collected within 2 days after birth and then after 1, 3, and 6 months. A saliva sample from the mothers was obtained 6 months after delivery. The parents were asked to complete a questionnaire on socioeconomic factors, lifestyle, and hygiene at baseline and throughout the study period. All samples were analyzed with 13 pre-determined bacterial probes using checkerboard DNA-DNA hybridization.

**Results:**

The groups were balanced at baseline concerning all relevant background factors. Gram-positive streptococci (*S. mitis, S. salivarius*) displayed the highest counts in both groups but a greater diversity was observed in the vaginally delivered group. *A. naeslundi, A. odontolytics, F. nucleatum* and *L. salivarius* were only detected among the vaginally delivered infants. The prevalence of *S. sanguinis, S. gordoni, R. denticariosa, and B. dentinum* increased by age in both groups but the prevalence was significantly lower in the C-section group (*p* < 0.05). There was a link between the mothers and their offspring’s concerning the salivary microbial profile.

**Conclusion:**

The microbial composition in saliva differs by the mode of delivery during the first six months of life.

## Background

The oral biofilm plays a significant role in health and disease [1]. The composition is highly individual and affected by factors such as genetics, diet, age and level of hygiene [2]. According to the hygiene hypothesis, the sequence and timing of exposure to a variety of bacterial strains early in life seem to be of importance for the gut colonization [3]. The mode of delivery and cessation of breastfeeding have been identified as major factors in gut microbiota maturation [4], and this seems applicable also to the oral cavity. Previous research has indicated that children delivered by caesarian section (C-section) exhibit a reduced microbial diversity in saliva in comparison with vaginally delivered children [5, 6]. Moreover, the oral microbial profile can discriminate breast-fed from formula-fed infants [7]. The long-term clinical importance of these findings is not fully clear but a reduced diversity in the guts correlates to increased risk of developing chronic disease such as asthma and allergy [8]. The information of lower diversity in C-sectioned infants is however mainly derived from cross-sectional studies and few longitudinal data are available. The aim of the present study was to analyze saliva samples from infants delivered with C-section through their first six months of life with respect to selected key bacteria. The results were compared with a group of vaginally delivered children and the null hypothesis was that no differences would be displayed between the groups.

## Methods

### Study design

The investigation employed a prospective case-control design and was ethically approved by the Regional Ethics Committee in Lund, Sweden (Dnr 2012/483) who has the authority to approve studies at Halland Hospital.

### Study group

The material consisted of 149 newborn infants delivered either vaginally (V; *n* = 96) or by C-section (CS; *n* = 53). The participants were consecutively enrolled among healthy volunteers that were born at Halland Hospital, Halmstad, Sweden, between April and December 2013. Both parents signed a consent form after thorough verbal and written information. Exclusion criteria were i) preterm children, ii) neonates in need of neonatal care, iii) infants with an unclear medical condition, and iv) families that planned relocation abroad after birth. The 6-month attrition rate was 20 % and the drop-outs with respect to the designated follow-ups are shown in Table 1.

**Table 1:** Participants and drop-out frequency in the two groups (CS = C-section; V = vaginal delivery)

Age	CS	V	Total	dropout rate
Newborn (1–2 days)	53	95	148	-
1 month	45	79	124	16 %
3 months	44	74	118	20 %
6 months	44	73	117	20 %

Values in the table denote number of children

### Saliva sampling

Samples of saliva were collected within 2 days after birth and then after 1, 3 and 6 months with aid of sterile cotton buds. At the first occasion, the parents were carefully instructed and shown how to collect the sample and transfer the top to a marked plastic tube. At the designated follow-ups, the parents received the test kit with a sterile containing a cotton bud and a plastic tube with an illustrated information sheet via surface mail. The parents were requested to collect the sample in the morning by soaking the cotton bud in saliva and immediately return it to the laboratory. When a sample was missing, the parents were reminded through a telephone call within 5 days. In a similar way, a saliva sample from the mothers was obtained 6 months after delivery. All samples were stored frozen until further analyses.

### Microbial method

The samples were processed with the checkerboard DNA-DNA hybridization technology according to Wall-Manning et al. [9]. DNA was extracted with mutanolysin and lysozyme as previously described [10] and the DNA quality was evaluated from the UV spectrum between 200 and 300 nm using a Gene Quant spectrophotometer (Pharmacia Biotech, Uppsala, Sweden). Whole genomic DNA probes of potential interest for oral health and disease were prepared from a panel of 13 bacterial species with strain designation according to the culture collection Oral Microbiology, Gothenburg, Sweden: *Actinomyces odontolyticus* G67; *Actinomyces naeslundii* 2466 (*A. oris*); *Bifidobacterium dentium* G174; *Fusobacterium nucleatum* 2865; *Lactobacillus casei* 3184; *Lactobacillus salivarius* 3830; *Rothia denticariosa* 1956; *Streptococcus gordonii* 2471; *Streptococcus mitis* 1770; *Streptococcus mutans* 2482; *Streptococcus salivarius* 2473; *Streptococcus sanguinis* 2478; *Veillonella parvula* G186. The bacterial counts were obtained by the percent-method in which the intensity of the signal (biomedical light units) was expressed as a fraction of a high standard sample (10^6^ cells) with aid of LumiImager Workstation (Boehringer Mannheim, Mannheim, Germany). The detection level was >10^4^ cells per mL sample.

### Questionnaire

At the inclusion, the parents were asked to complete a 20-item questionnaire on the family characteristics, socio-economy, lifestyle factors and hygiene habits. A further questionnaire on breeding, feeding, tooth brushing habits and prescribed antibiotics was attached to the follow-up mailings and returned together with the samples.

### Statistical methods

All data were processed with the IBM-SPSS software (version 22.0; Chicago IL, USA). Differences in percentage distributions between the groups were calculated with chi-square tests and correlations between the bacterial counts in mothers and their offspring’s were expressed as Spearman correlation coefficient. We considered *p*-values less than 0.05 as statistically significant.

## Results

The groups were balanced at baseline concerning all relevant background factors, except for the water break (for exact details, see Table 2). The proportion of girls was somewhat higher in the CS group but the difference was not statistically significant. The self-reported feeding routines and the use of pacifiers are shown in Table 3. Infant formulas were more common in CS group compared with the V group during the first 6 months but the difference did not reach statistical significance. The frequency of prescribed antibiotics to the mothers during the first six months after delivery was similar (<15 %) in both groups. The total number of cells detected from the V samples was on average higher than the CS samples at all sampling occasions: birth: 0.7 vs. 0.5x10^6^; 1 month: 1.3 vs. 0.9x10^6^; 3 months: 1.6 vs 0.9x10^6^; 6 months: 1.5 vs 0.9x10^6^ cells. The prevalence of the selected bacteria is summarized in Table 4. A greater diversity was observed in the V group. For example, *A. naeslundi*, *A. odontolytics*, *F. nucleatum* and *L. salivarius* were only detected among the vaginally delivered infants. The prevalence of *S. sanguinis*, *S. gordoni*, *R. denticariosa* and *B. dentinum* increased with age in both groups but the prevalence was significantly lower in the CS group (*p* < 0.05). *S. mitis* and *S. salivarius* displayed the highest counts in both groups at all samplings but the counts were not associated with the mode of delivery. There was a statistically significant correlation between the mothers and their offspring’s concerning the salivary profile six months after the delivery with respect to five strains in the CS group and eight strains in the V group (Table 5).

**Table 2:** Baseline family characteristics in the two groups (CS = C-section; V = vaginal delivery)

Variable	CS	V
Sex (girls/boys)	57/43	44/56
Water break (yes)	29	100^a^
Mother born in Sweden (yes)	91	91
Mother’s education, university (yes)	64	60
Mother smoking (yes)	0	4
Prescribed antibiotics (mother)	4	4
Siblings (>2)	6	6

Values in the table denote percent.

^a^statistically significant difference between the groups (*p* < 0.05, chi-square test)

**Table 3:** Self-reported data from the questionnaires distributed 1, 3 and 6 months after vaginal delivery (V) or C-section (CS)

Variable	1 month	3 months	6 months
	CS/V	CS/V	CS/V
Breast feeding (yes)	89/95	71/84	55/69
Infant formula (yes)	47/32	51/35	50/44
Introduced food-tasting (yes)	0/0	0/4	72/83
Pacifier (yes)	80/83	82/77	85/71
Teeth present (yes)	0/0	2/0	30/32
Oral cleaning (yes)	0/0	9/3	30/26
Antibiotics (yes, infant)	0/3	0/1	0/1
Antibiotics (yes, mothers)	9/5	5/4	0/3

Values in the table denote percent

**Table 4:** Prevalence (≥10^4^ cells) of the selected strains at birth and designated time intervals in children delivered vaginally (V) or with C-section (CS)

Strain	birth	1 month	3 months	6 months
	CS/V	CS/V	CS/V	CS/V
*A. naeslundi*	0/5	0/0	0/2	0/2
*A. odontolyticus*	0/12^a^	0/32^a^	0/30^a^	0/32^a^
*B. dentinum*	5/32^a^	13/61^a^	8/49^a^	16/63^a^
*F. nucleatum*	0/2	0/11	0/10	0/14^a^
*L. casei*	0/2	0/0	0/0	0/0
*L. salivarius*	0/0	0/0	0/0	0/3
*R. denticariosa*	0/12^a^	3/22^a^	10/21	8/39^a^
*S. gordoni*	3/15^a^	10/19	13/32^a^	13/38^a^
*S. mitis*	73/84	85/94	90/94	93/96
*S. mutans*	53/76^a^	65/88^a^	58/88^a^	68/92^a^
*S. salivarius*	28/36	45/67^a^	53/71	48/70
*S. sanguinis*	0/35^a^	5/44^a^	10/47^a^	13/53^a^
*V. parvula*	0/0	3/6	5/6	8/24

Values in the table denote percent

^a^statistically significant difference between the groups (*p* < 0.05, chi-square test)

**Table 5:** Oral colonization of selected bacteria in mothers and their infants, 6 months after vaginal delivery (V) or C-section (CS)

Strain	CS	V
*A. naeslundi*	NS	NS
*A. odontolyticus*	NS	0.43
*B. dentinum*	0.54	0.28
*F. nucleatum*	NS	NS
*L. casei*	NS	NS
*L. denticariosa*	NS	0.45
*L. salivarius*	NS	NS
*S. gordonii*	0.48	0.36
*S. mitis*	0.59	0.32
*S. mutans*	0.85	0.36
*S. salivarius*	0.71	0.48
*S. sanguinis*	NS	0.56
*V. parvula*	NS	NS

The values in table denote Spearman two-tailed correlation coefficient. *NS* Not statistically significant

## Discussion

The results of this relatively large study lend further support to previous findings that the composition of the oral biofilm is affected by the mode of delivery [5, 6, 11] with a lower diversity among infants delivered with C-section. Hence, the null hypothesis could be rejected. Our results must however be considered with some caution for a number of reasons. First of all, the composition of the early salivary microbiota may be influenced by a number of factors other than the mode of delivery, such as peri-delivery treatment with antibiotics, diet [12], feeding practices [7, 13], use of pacifiers [14], family income [11, 13], and tooth brushing habits [15]. In our study, the CS and the vaginally delivered groups were similar with respect to socio-economy, family size, maternal education level, and feeding behavior so we consider the risk for confounders as small. Even the prescription of antibiotics to the mothers and their off-springs was equal according to the questionnaires, albeit a certain underreporting may be suspected. Secondly, the checkerboard DNA-DNA hybridization technique has its strengths and limitations; the main advantage is that the method permits enumeration of large numbers of species in large numbers of samples and hence, considered as a useful tool for the enumeration of bacterial species in complex microbial systems [16]. Among the limitations, possible cross-reactions and varying reproducibility for different strains has been discussed [17]. Thirdly, it is possible that the sampling procedure may have caused a greater variability in results than the laboratory procedure as such. These shortcomings, together with the relatively high detection level made us to quantify the bacterial data according to the percent method as being the most reliable alternative [17]. It is however important to underline that the prevalence values reported here was based on a threshold level of approximately 10^4^ cells; the selected strains could very well be present in the samples but not in enough amounts to be detected. Thus, the next natural step would therefore be to extend the mapping to include the entire oral microbiome. A fourth possible bias was the relatively high dropout rate during the course of the study, and we can only speculate on its possible influence on the results.

The intra-family correlation of the oral microflora with special reference to mother-child and mutans streptococci acquisition has been described several times before [18–21]. Interestingly, Ruiz-Rodriguez and co-workers [22] failed to demonstrate a mother-child relationship for *S. mutans* with conventional culturing but their sample was small and *S. mutans* was very rarely detected (2 %) among the infants at the age of 150 days. In the present study, the majority of all children in the CS group and almost all in the V group harbored *S. mutans* at 6 months of age and apart from the different sampling and analytical techniques, we have no explanation to these contrasting findings. Nevertheless, the clear mother-child correlation for 8 of the 13 stains in the V group was interesting but needs to be further investigated with for example 16 s rDNA amplification and sequencing.

The question for how long a delivery-dependent difference in oral colonization prevails, and whether this may affect dental health later in life, remains open. There is however fair evidence that an early acquisition of oral mutans streptococci in general is associated with an increased risk for caries development [23, 24]. Furthermore, there are reports suggesting that children delivered with C-section have an elevated risk for childhood asthma [25] and more dental caries at the age of 3–5 years [26, 27]. This possible relationship needs however to be verified in larger settings and in prospective studies covering the young permanent dentition.

## Conclusions

Within the limitations of the present study, it was concluded that mode of delivery plays a role in the composition of the oral biofilm during the first 6 months of life. Vaginally delivered children appear to have a greater diversity than C-section children and a closer similarity with their mothers. Further studies to investigate a possible long-term influence on oral health seem warranted.

## Abbreviation

CS: Caesarian section
